# Bilateral Retrocerebellar Arachnoid Cysts Presenting as Peripheral Vertigo: Case Report and Literature Review

**DOI:** 10.7759/cureus.9139

**Published:** 2020-07-11

**Authors:** Matthew D Lee, Nathan Ziman, Juan Deleija

**Affiliations:** 1 Internal Medicine, Baylor College of Medicine, Houston, USA

**Keywords:** arachnoid, cyst, retrocerebellar, bilateral, vertigo, case, report

## Abstract

Bilateral retrocerebellar arachnoid cysts are exceedingly rare. We report a case of a 38-year-old woman, who presented with progressive vertigo and was found to have bilateral retrocerebellar arachnoid cysts. The patient’s clinical presentation was most consistent with benign positional peripheral vertigo, while the cysts were thought to be incidental findings. We review the literature on bilateral retrocerebellar arachnoid cysts and discuss their management.

## Introduction

Intracranial arachnoid cysts are found in approximately 1% of the adult population, have a higher prevalence in men, are usually unilateral and asymptomatic, and most commonly occur in the middle fossa [1,2]. Bilateral retrocerebellar arachnoid cysts are extremely rare with only three cases reported in the literature [3-5]. We present another case of this rare pathology.

## Case presentation

A 38-year-old woman presented to the hospital with progressive lightheadedness and vertigo. Her past medical history includes a gunshot wound to the face that occurred 12 years prior to admission with residual shrapnel and multiple reconstructive surgeries. Since her gunshot wound, she developed monthly episodes of transient lightheadedness and dizziness. In the week prior to admission, these episodes occurred multiple times daily, affected her balance, and were associated with double vision. During an episode, she experienced a fall upon standing which prompted presentation to the hospital. She denied nausea, vomiting, and a clear association with headaches.

**Figure 1:**
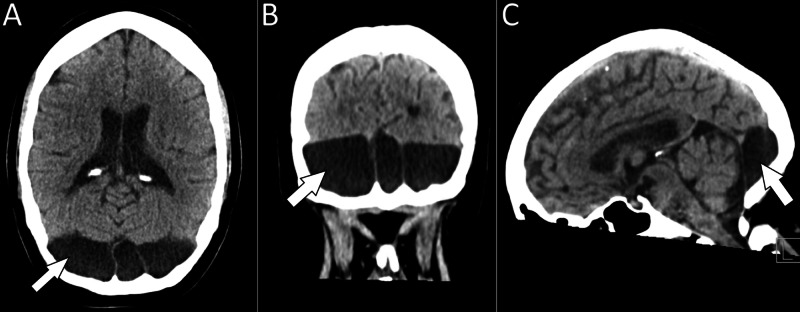
CT of the head shows retrocerebellar hypodensities with septations, consistent with arachnoid cysts bilaterally and in the midline, with mild mass effect on the cerebellum in (A) axial, (B) coronal, and (C) sagittal views.

Physical examination revealed a positive Romberg sign, unbalanced gait, negative HINTS (Head Impulse, Nystagmus, Test of Skew) exam, and positive Dix-Hallpike test on the left. Laboratory tests were unremarkable except for mild iron-deficiency anemia with a hemoglobin of 11 g/dL. Electrocardiogram was normal. CT of the head revealed bilateral retrocerebellar arachnoid cysts exerting mild mass effect on the cerebellum (Figure 1).

**Figure 2:**
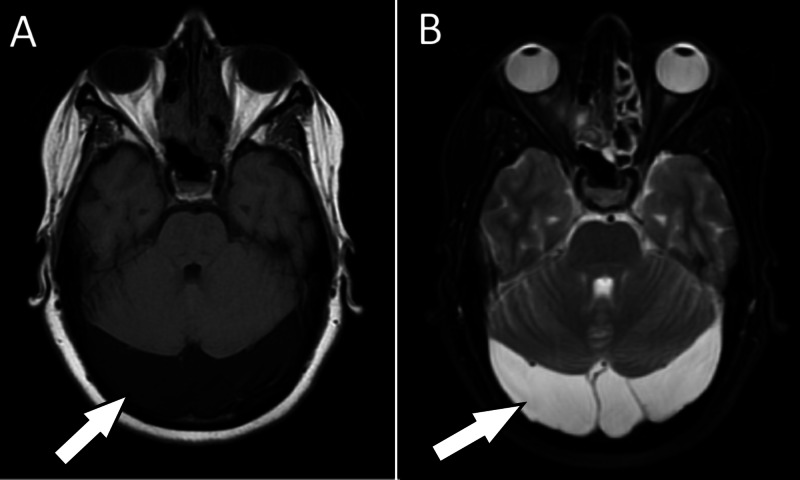
MRI of the brain without contrast shows bilateral retrocerebellar arachnoid cysts with mild mass effect on the cerebellum, which appear (A) hypointense on T1-weighted imaging and (B) hyperintense on T2-weighted imaging.

Given the patient’s history of residual shrapnel in the face, skull radiographs were obtained and confirmed punctate metallic densities in the right cheek. Because these fragments were far from the orbits or cranium, MRI of the brain was pursued. MRI without intravenous contrast redemonstrated the bilateral retrocerebellar arachnoid cysts and ruled out other pathology (Figure 2). The patient’s clinical presentation was thought to be most consistent with benign positional peripheral vertigo. The patient declined the Epley maneuver but reported improvement with Brandt-Daroff exercises and meclizine. 

## Discussion

The etiology of arachnoid cysts remains unclear. Congenital arachnoid cysts are thought to form during embryonic development from splitting or duplication of the arachnoid membrane, which then fills with fluid and forms a cyst [2]. Intracranial arachnoid cysts are found in approximately 1% of the adult population and can be found in pediatric and adult patients of all ages [1,2]. Arachnoid cysts are more prevalent in males than females with one large study reporting a prevalence of 1.8% in males compared to 1.1% in females [1]. Most arachnoid cysts are supratentorial and unilateral with the most common location being the left middle fossa [1,2]. While most are asymptomatic and incidental findings that do not require specific treatment, arachnoid cysts are symptomatic in about 5% of cases [1]. Almost all arachnoid cysts remain stable in size [1,2]. In rare cases, cysts may enlarge and cause hydrocephalus or other symptoms based on location [1].

The differential diagnosis of retrocerebellar arachnoid cysts includes mega cisterna magna, Dandy-Walker malformation, vermian-cerebellar hypoplasia, epidermoid cysts, cystic tumors, non-neoplastic cysts, subdural hygroma, and chronic subdural hemorrhage [2,6,7]. Imaging is key to differentiating between these diagnoses.

In the present case, the patient’s history and physical exam findings favored benign positional peripheral vertigo. The mild mass effect the arachnoid cysts exerted on the cerebellum was not considered significant enough to result in her symptoms. MRI ruled out other etiologies, such as brainstem infarction and demyelinating disease. Furthermore, the patient’s improvement with Brandt-Daroff exercises and meclizine also supported our conclusion that the arachnoid cysts were not the cause of her symptoms. By the time of this writing, three months after discharge, she had not presented to a clinic or hospital in our system with recurrent symptoms.

Bilateral retrocerebellar arachnoid cysts are extremely rare with only three other cases reported in the literature [3-5].

1. Jeevanandham et al. report the case of a 12-year-old girl, who presented with one year of progressive bilateral visual impairment, short stature, and cheek fullness [3]. Fundoscopy revealed bilateral optic nerve atrophy. Imaging revealed multiple expansile lytic lesions in the mandible, consistent with cherubism, as well as incidental bilateral retrocerebellar arachnoid cysts. The authors do not report on the patient’s management or outcome.

2. Killeen et al. describe the case of a 33-year-old man, who presented with several weeks of headache, dizziness, fatigue, intermittent dysarthria, bilateral upper and lower limb paresthesias, and drop attacks [4]. Imaging revealed bilateral retrocerebellar arachnoid cysts and cerebellar tonsillar ectopia. The patient underwent bilateral burrhole drainage, which improved his symptoms. However, nine months later, the patient’s symptoms recurred, and the right cyst had reaccumulated, requiring repeat drainage. The patient’s symptoms resolved, and he remained asymptomatic 18 months later.

3. Kuratsu et al. report the case of a two-year-old boy, who presented with macrocephaly [5]. Imaging revealed a large multilocular arachnoid cyst extending from the bilateral paracollicular to bilateral retrocerebellar regions, as well as obliteration of the fourth ventricle. The patient underwent placement of a shunt between the retrocerebellar cyst and peritoneal cavity, which resulted in decreased mass effect and visualization of the fourth ventricle. The authors do not report on the patient’s long-term outcome.

Most arachnoid cysts are benign, and neurosurgical treatment is reserved for those with severe, intractable symptoms or neurologic damage that can be clearly attributed to the cysts. When arachnoid cysts cause hydrocephalus or are thought to unequivocally cause signs and symptoms of neurological compression or damage, neurosurgical evaluation is warranted. Surgical interventions aim to decrease the pressure the cyst exerts on surrounding structures or to eliminate an obstruction of cerebrospinal fluid. Surgical techniques for symptomatic cysts include craniotomy with cyst excision or fenestration, endoscopic fenestration, and shunting [8]. A 2019 meta-analysis found that these treatments improved patient outcomes with no significant difference between them.

## Conclusions

We present a rare case of bilateral retrocerebellar arachnoid cysts. The patient presented with progressive vertigo consistent with benign positional peripheral vertigo, while the cysts were likely incidental. The patient improved with medical management. In cases where severe symptoms are attributed to arachnoid cysts, neurosurgical treatment may be considered.

## References

[1] Al-Holou WN, Terman S, Kilburg C, Garton HJL, Muraszko KM, Maher CO (2013). Prevalence and natural history of arachnoid cysts in adults. J Neurosurg.

[2] Cincu R, Agrawal A, Eiras J (2007). Intracranial arachnoid cysts: current concepts and treatment alternatives. Clin Neurol Neurosurg.

[3] Jeevanandham B, Ramachandran R, Dhanapal V, Subramanian I, Sai V (2018). Orphan disease: cherubism, optic atrophy, and short stature. Indian J Radiol Imaging.

[4] Killeen T, Tromop-Van-Dalen C, Alexander H, Wickremesekera A (2013). Bilateral retrocerebellar arachnoid cysts exerting mass effect and associated with cerebellar tonsillar ectopia in an otherwise healthy Adult. Neurol Med Chir.

[5] Kuratsu J, Matsukado Y, Kodama T (1983). Large infratentorial multilocular arachnoid cyst. Pediatr Neurosurg.

[6] Kollias SS, Ball WS, Prenger EC (1993). Cystic malformations of the posterior fossa: differential diagnosis clarified through embryologic analysis. RadioGraphics.

[7] Dutt SN, Mirza S, Chavda SV, Irving RM (2002). Radiologic differentiation of intracranial epidermoids from arachnoid cysts. Otol Neurotol.

[8] Hayes MJ, TerMaath SC, Crook TR, Killeffer JA (2019). A review on the effectiveness of surgical intervention for symptomatic intracranial arachnoid cysts in adults. World Neurosurg.

